# Characterization of Sleep Disturbances in Children and Adolescents with Down Syndrome and Their Relation with Cognitive and Behavioral Features

**DOI:** 10.3390/ijerph18095001

**Published:** 2021-05-09

**Authors:** Elisa Fucà, Floriana Costanzo, Laura Celestini, Alessandra Mandarino, Stefano Vicari

**Affiliations:** 1 Child and Adolescent Neuropsychiatry Unit, Department of Neuroscience, Bambino Gesù Children’s Hospital, IRCCS, 00165 Rome, Italy; elisa.fuca@opbg.net (E.F.); lau.celestini@gmail.com (L.C.); alessandra.mandarino@opbg.net (A.M.); stefano.vicari@opbg.net (S.V.); 2 Department of Life Science and Public Health, Università Cattolica del Sacro Cuore, 00168 Rome, Italy

**Keywords:** sleep, mental health, trisomy 21, childhood, adolescence, externalizing symptoms

## Abstract

Despite sleep disturbances are common among youths with Down syndrome (DS), the cognitive and behavioral features associated with sleep problems have not yet been studied extensively. The present study investigated the presence of sleep disturbances in a group of children and adolescents with DS and their cognitive and behavioral correlates. Seventy-one children and adolescents with DS underwent a neuropsychological evaluation, whereas parents completed questionnaires for the screening of the child’s sleep, emotional and behavioral problems. We found no association between sleep disturbances and sex, nonverbal IQ, nor adaptive abilities. However, we found that age was positively associated with disorders in initiating and maintaining sleep (DIMS) and disorders of excessive somnolence (DOES), while body mass index was related with DOES. We also detected a relationship between visual-motor integrations and DIMS, as well as multiple associations between sleep disturbances and psychopathological and behavioral problems, mainly externalizing symptoms. The present study provided a detailed characterization of sleep problems in relation to several features of youths with DS. The proper identification of sleep disturbances profile in the DS population could support the process of clinical evaluation, in particular for psychopathological aspects.

## 1. Introduction

Caused by a third copy of chromosome 21, Down syndrome (DS) is the most frequent genetic cause of intellectual disability, with an estimated birth prevalence of 14 per 10,000 live births [1,2]. Individuals with DS exhibit peculiar dysmorphic features as brachycephaly, small ears, epicanthic folds, flat nasal bridge, small mouth with protruding large tongue, mandibular hypoplasia, relatively large and medially positioned tonsils, and macroglossia [3,4]. DS phenotype also involves many body systems: people with DS exhibit a high number of comorbidities and complex medical conditions, as cardiac complications, airway, pulmonary, and hearing problems, hematologic, autoimmune, oncologic, and musculoskeletal disorders [5]. These clinical features are not present in every child and, if present, they widely differ among individuals.

Such a complex phenotype makes individuals with DS prone to sleep disturbances. In particular, obstructive sleep apnea is frequent in children with DS, with a prevalence rate ranging between 22 and 79% [6,7,8,9], compared to 1.1–3.5% in typically developing (TD) children [10]. Moreover, higher rates of other sleep disorders have been reported in children with DS [11], such as daytime tiredness (70%) and bedtime resistance (25%), restlessness during sleep (58%), night waking (40%), not falling asleep in own bed (33%), and bedwetting (26%). Similarly, MacCrosain and Byrne [12] (2009) showed higher scores for children with DS on all the subscales of the Children’s Sleep Habits Questionnaire compared to published data for TD children aged 4–10, indicating severer bedtime resistance, sleep onset delay, sleep duration, sleep anxiety, night waking, parasomnias, sleep-disordered breathing, and daytime sleepiness. A more recent study [13] confirmed significantly higher bedtime resistance, sleep anxiety, night waking, daytime sleepiness, and total disturbance score in DS children relative to TD controls. Similar findings were obtained by the use of objective measures, as actigraphy and polysomnography [14,15,16]. Sleep disturbances in DS arise at an early age, and they may persist with increasing age [7,17].

The associations between sleep quality and medical comorbidities, cognitive deficits, and behavioral characteristics in children with DS have also been investigated. Research on the link between sleep disturbances and obesity in the DS population did not provide consistent results. Some findings documented the negative effects of obstructive sleep apnea on the cardiovascular system, with studies reporting increased sympathetic activity, elevated blood pressure and heart rate, and decreased autonomic control of blood pressure and heart rate [18]. These consequences are particularly impactful for individuals with DS, given their abovementioned proneness to a variety of medical comorbidities. Moreover, Fitzgerald and colleagues [19] failed to detect an association between body mass index (BMI) and obstructive sleep apnea in children with DS, whereas a subsequent retrospective case by Shires and colleagues [20] reported such association.

As concerns cognitive aspects, sleep disorders in children with DS have been associated with lower verbal IQ [21,22], worse verbal fluency [23], decreased expressive communication [9,24], and poorer executive functioning [21,25]. Finally, sleep disturbances in DS are related to a range of emotional and behavioral problems as hyperactivity/impulsivity, conduct problems, and anxiety [25,26,27,28].

The characterization of sleep disturbances and their associated features in children with DS is essential since adequate sleep is crucial for learning, attention, memory, and executive functioning [29,30]. Thus, sleep problems may worsen learning and behavioral problems in children with DS. In fact, it has been well documented how sleep disturbances affect their school performance, limit social interactions, interfere with the accomplishment of daily activities, and affect the quality of life in their parents [17,25,31,32,33,34]. Because of the prevalence among people with DS, sleep breathing disorders and associated neuropsychological and psychopathological features have been largely investigated; however, less is known about the neuropsychological and psychopathological correlates of other sleep disturbances. As highlighted by a recent review on this topic, the impact of different kinds of sleep disorders in children with DS still requires widely researched and well-designed studies to better characterize such relationships [35].

The aim of the current study is to provide an in-depth understanding of sleep disturbances and their correlates in children and adolescents with DS. In particular, we wanted to: (i) investigate the prevalence of different kinds of sleep disturbances in youths with DS; (ii) provide a detailed description of cognitive and behavioral features associated with specific profiles of sleep problems in such population.

## 2. Materials and Methods

### 2.1. Participants

Seventy-one youths with DS (50 boys, 21 girls) ranging in age from 3.4 to 18 years, with a mean age of 9.92 ± 3.92 years, were included in the study. Participants consisted of 15.5% children in preschool age, 59% children aged 6–11, and 25.5% youths aged 12–18. The mean BMI was 19.43 ± 3.74. The mean IQ was 55.27 ± 8.77. Participants consisted of 90.2% Caucasian, 7% Hispanic, and 2.8% Asian. Selection criteria included, besides the diagnosis of DS based on the analysis of the karyotype, the age ranging between 3 and 18 years. Exclusion criteria were as follows: the ascertained presence or the clinical suspect of neurological conditions; language barrier hampering questionnaire compilation by parents.

### 2.2. Procedure

Data were retrospectively collected from a file review of patients with DS referred for a clinical evaluation at the Child and Adolescent Neuropsychiatry Unit of the Bambino Gesù Children’s Hospital in Rome between November 2019 and September 2020. Children and adolescents with DS underwent a neuropsychological evaluation, whereas parents completed parent-report measures, such as the Sleep Disturbance Scale for Children (SDSC) [36] and psychopathological questionnaires regarding their child.

### 2.3. Measures

#### 2.3.1. Screening for Sleep Disorders

Sleep disturbances were assessed by means of SDSC [36,37,38], a questionnaire that has demonstrated through validation an adequate level of internal consistency, test-retest reliability, and availability of normative data. The SDSC explores the presence of sleep disorders during the previous six months and contains 26 items with Likert scale values of 1–5. The questionnaire consists of 26 items subdivided into six sleep disorder subscales: disorders in initiating and maintaining sleep (DIMS), sleep breathing disorders (SBD), disorders of arousal (DA), sleep–wake transition disorders (SWTD), disorders of excessive somnolence (DOES), and sleep hyperhidrosis (SHY). According to Bruni and collaborators (1996), internal consistency is high in controls (0.79) and at a satisfactory level in clinical subjects (0.71). Correlations between subscales and the total score are unacceptable to acceptable (*r* = 0.45–0.77) for children without sleep disorders and lower for children with sleep disorders (*r* = 0.31–0.69). The SDSC total score has demonstrated suitable concurrent validity with diagnosed sleep disorders (insomnia, hypersomnia, respiratory disturbances during sleep, and parasomnias). The subscales have demonstrated low inter-correlations. The sum of scores provides a total sleep score with a possible range from 26 to 130; a T score of more than 70 was regarded as pathological. The results were classified into pathological, borderline, or normal, using a cutoff value according to the validation criteria of the test.

#### 2.3.2. Neuropsychological Evaluation

Cognitive development was tested by the Leiter-3 [39], which provides a nonverbal measure of intelligence and assesses the ability to reason by analogy, by matching and perceptual reasoning in general, irrespective of language and formal schooling for individuals ages 3–70. The nonverbal IQ obtained through this test is based on four subtests: Figure Ground, Form Completion, Classification and Analogies, and Sequential Order. The Griffiths III [40] was administered only in few cases (8 participants) when the child failed to complete the Leiter-3 because of his/her reduced attentional resources. The development quotient obtained from Griffiths III is based on five subscales: Language and Communication Subscale, Eye and Hand Coordination Subscale, Personal, Social Emotional Subscale, and Gross Motor Subscale.

The Beery-Buktenica Developmental Test of Visual-Motor Integration for individuals ages 3–18 (Beery VMI) [41] was used to assess visual-motor integration. The Beery VMI is comprised of drawings of geometric designs that increase in difficulty; participants are asked to observe the geometric designs and copy them with paper and pencil. Scoring criteria are based on the accuracy with which the designs were copied: higher scores indicate higher visual-motor ability. Raw scores are converted to standard scores according to the participant’s age.

#### 2.3.3. Evaluation of Adaptive Abilities

To assess the presence of impairments in adaptive behaviors necessary for socialization, communication, and daily functioning, we used the Adaptive Behavior Assessment System II (ABAS-II) [42], a parent/primary caregiver questionnaire. ABAS-II consists of eleven skill areas organized into three general domains: conceptual, practical, and social. The composite and domain scores are standard scores with a norm-referenced mean of 100 and a standard deviation of 15.

#### 2.3.4. Evaluation of Psychopathological Symptoms and Behavioral Problems

The Child Behavior Checklist (CBCL) [43,44] was used to assess emotional and behavioral problems. For preschool children, we used the CBCL for ages 1.5 to 5, which consists of 100 problem items. There are seven syndrome scales: Emotionally Reactive, Anxious/Depressed, Somatic Complaints, Withdrawn, Sleep Problems, Attention Problems, and Aggressive Behavior. The summary profile contains the Internalizing, Externalizing, and Total Problems scales. The version for school-aged youths consists of a 118-items scale with syndromes scales (Anxious/Depressed; Withdrawn/Depressed; Somatic Complaints; Social Problems; Thought Problems; Rule-Breaking Behavior; Attention Problems; Aggressive Behavior), a Total Problem Score, and two broad-band scores (Internalizing Problems and Externalizing Problems). The analysis of CBCL scores was performed for the scales overlapping between the two versions of the questionnaire, namely: Anxious/Depressed, Somatic Complaints, Attention Problems, Aggressive Behavior, Internalizing Problems, Externalizing Problems, and Total Problems.

Conners’ Rating Scales Long Version Revised (CPRS) [45]. This 80-items questionnaire was completed by parents to obtain a measure of attention-deficit/hyperactivity disorder criteria for hyperactivity and inattention. The questionnaire contains scales A through N (oppositional; inattention; hyperactivity; anxiety; perfectionism; social problems; psychosomatic problems; ADHD index; CGI: restlessness; CGI: emotional instability; CGI: total; DSM-IV: inattention; DSM-IV: hyperactivity/impulsivity; DSM-IV: total). Scores can then be converted to a T score. CPRS was administered in a subsample of 50 youths.

The Aberrant Behavior Checklist (ABC) [46] is a caregiver rating scale designed to assess the severity of core symptoms and comorbid emotional and behavioral problems for several neurodevelopmental and genetic disorders, including DS [47,48]. The scale consists of 58 questions with five subscales: Irritability/Agitation/Crying; Lethargy/Social Withdrawal; Stereotypic Behavior; Hyperactivity/Noncompliance; Inappropriate Speech.

The Repetitive Behavior Scale-Revised (RBS-R) [49] is an empirically derived clinical rating scale including 43-items in a parent-completed questionnaire. Items are grouped into six subscales: (1) Stereotyped Behavior; (2) Self-Injurious Behavior; (3) Compulsive Behavior; (4) Ritualistic Behavior; (5) Sameness Behavior; and (6) Restricted Interests Behaviors.

#### 2.3.5. Evaluation of Maternal Stress

The Parenting Stress Index-Short Form (PSI-SF) [50] was used to measure maternal stress. The PSI-SF consists of 36 questions, which capture three domains: parental distress, parent–child dysfunctional interaction, and difficult child. The Parental Distress (PD) subscale evaluates aspects that may affect parenting activities, such as limited social support and parental conflict with the partner. The Difficult Child (DC) subscale assesses parenting challenges related to a child’s self-regulation or behavioral difficulties. The Parent–Child Dysfunctional Interaction (P-CDI) subscale is designed to assess unsatisfactory parent–child interactions. The sum of all questions results in the Total Stress score.

### 2.4. Statistical Analysis

Statistical analyses were performed using the Statistical Package for the Social Sciences, version 13.0 (IBM Corp, Armonk, NY, USA). Descriptive statistics were used to analyze the demographic and clinical characteristics of the whole sample. Pearson and Spearman’s correlation analyses were used to evaluate the relationship between sleep disturbances and the identified variables. Given the recognized pattern of changes in emotional and behavioral functioning across age in the DS population [51], we performed partial correlation analyses to investigate the relationship between sleep problems, psychopathological, and behavioral features in our sample while controlling for age. A Bonferroni correction for multiple comparisons was applied, and according to the number of comparisons, a different p-value was considered statistically significant.

## 3. Results

### 3.1. Distribution of Sleep Disturbances

**Figure 1:**
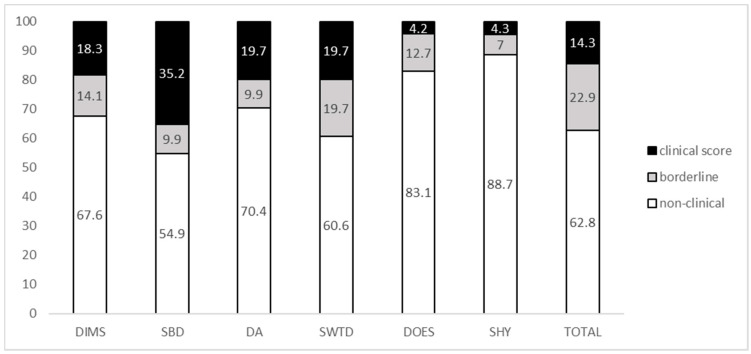
Distribution of sleep disturbances in children and adolescents with DS (percentage). DIMS: disorders in initiating and maintaining sleep; SBD: sleep breathing disorders, DA: disorders of arousal, SWTD: sleep–wake transition disorders, DOES: disorders of excessive somnolence; SHY: sleep hyperhidrosis.

The distribution of sleep disturbances in our sample, assessed by the SDSC questionnaire, is summarized in Figure 1. SBD was the most represented sleep problem, with 35.2% of clinical scores registered, followed by DA and SWTD (19.7% clinical scores each), DIMS (18.3% clinical scores), and DOES and SHY (4.2% clinical scores each). A total of 14.5% of total scores at SDSC was in the clinical range and 22.9% in the borderline range, whereas 62.8% of participants registered scores in the non-clinical range.

### 3.2. Association with Individual Features, Neuropsychological Variables, and Adaptive Profile

We did not detect any association between SDSC scales and sex nor adaptive abilities assessed by ABAS-II. Conversely, a significant association between BMI and DOES emerged (*r* = 0.502, *p* = 0.002), as well as between age and DIMS (*r* = 0.258, *p* = 0.030) and DOES (*r* = 0.263, *p* = 0.027). As concerns neuropsychological variables, we failed to detect any association with IQ, but a significant negative association between visual-motor integration standard score and DIMS emerged (*r* = −0.330, *p* = 0.024).

### 3.3. Association with Psychopathological, Behavioral Features, and Maternal Stress

**Figure 2:**
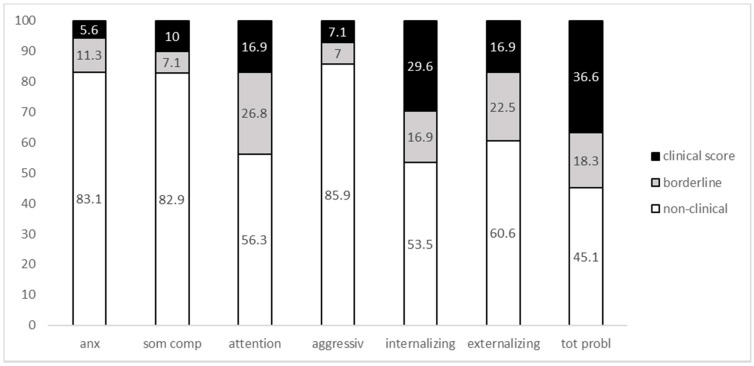
Distribution of scores in CBCL scales (percentage). Anx. Anxious/depressed; Som. Comp. Somatic Complaints; Aggress. Aggressive Behavior; Intern. Internalizing Behavior; Extern. Externalizing Behavior; Tot Probl. Total Problems.

An overview of the distribution of CBCL scores in our sample is provided in Figure 2. The highest percentage of clinical scores was observed for the Total Problems scale (36.6%), followed by Internalizing Problems (29.6%), Attention and Externalizing Problems (16.9% each), Somatic Complaints (10%), Aggressive Behavior (7.1%) and Anxious/Depressed (5.6%).

The strongest associations emerged between DIMS and CBCL scales related to the externalizing domain (i.e., Attention, Externalizing Behavior, and Total Problems), as well as between SWTD and Somatic Complaints and Attention subscales. DOES highly correlated with both internalizing domain (Anxious/Depressed, Somatic Complaints subscales) and Externalizing Behavior scale. Detailed results are reported in Table 1.

To further investigate the relationship between externalizing symptoms and sleep disturbances, we used the CPRS. Results are summarized in Table 2. DIMS and Total scores significantly correlated with almost all CPRS scales, whereas no association between SBD and CPRS scales has been detected. We found weak correlations between DA and the following scales: Cognitive problems/inattention, Perfectionism, Psychosomatic, ADHD index, Global index—Restless-impulsive, CPRS Global index, DSM-IV Hyperactive/impulsive, DSM-IV Total. STWD was strongly associated with the Global index—Restless-impulsive, Emotional Lability, and CPRS Global index scales, whereas DOES was highly correlated with Anxious and DSM-IV Hyperactive/impulsive scales. SHY was not associated with any CPRS scale, except a weak association with the Emotional Lability scale.

In order to explore the association between sleep disturbances and behavioral problems, we used the ABC. Again, the highest correlations have been detected between DIMS and nearly all the ABC subscales. Detailed results are reported in Table 3.

As concerns the association with repetitive behavior, assessed through RBS, significant correlations emerged between DIMS and the following scales: stereotyped behavior (r = 0.376; *p* = 0.008), sameness (r = 0.292; *p* = 0.042), and RBS Total score (r = 0.310; *p* = 0.030), as well as between DA and the following scales: stereotyped behavior (r = 0.343; *p* = 0.16), ritualistic behavior (r = 0.319; *p* = 0.025) and Total score (r = 0.310; *p* = 0.030). We also detected significant associations between DOES and compulsive behavior subscale (r = 0.316; *p* = 0.027) and between Total score and stereotyped behavior (r = 0.375; *p* = 0.008), compulsive behavior (r = 0.287; *p* = 0.046) and RBS Total Score (r = 0.327; *p* = 0.022). However, no correlation survived after Bonferroni correction (*p* ≤ 0.001).

The correlation analysis between scales of SDSC and PSI revealed a significant association between DIMS and DC (r = 0.346, *p* = 0.009).

## 4. Discussion

In this study, we examined relations between sleep disturbances and individual and clinical features in a sample of children and adolescents with DS. With this aim, we used a large range of neuropsychological and psychological tests, including measures to evaluate cognitive abilities, visual-motor integration, psychopathology, and behavioral problems. We found that sleep disturbances were not associated with sex, nonverbal IQ, nor adaptive abilities. Considering the individual features, we demonstrated a positive correlation between age, DISM, and DOES, as well as between DOES and BMI. Considering the neuropsychological and clinical features, we reported a relationship between visual-motor integrations and DIMS and multiple associations between several SDSC subscales and psychopathological and behavioral problems, mainly externalizing symptoms.

### 4.1. Prevalence of Sleep Disturbances and the Role of Individual and Neuropsychological Features

The distribution of sleep disturbances in our sample is consistent with the prevalence previously reported in the literature in a study using SDSC in youths with DS [52], in particular for DIMS, DOES, SHY, and SDSC Total score. However, some discrepancies emerged in the percentage of SBD and DA, with a higher percentage of clinical scores observed in our sample. Such differences could be partially related to the wider age range of participants included in our study. It has been documented, indeed, that the prevalence of moderate to severe SBD in DS and, in particular, obstructive sleep apnea is higher at a younger age [9]. This developmental trajectory has also been reported for DA in both DS [8] and TD populations [53]. A modest but significant association with age emerged for DIMS and DOES in our sample. This is in line with previous literature indicating that some kinds of sleep problems seem to increase with age in DS [11]. However, research exploring sleep disturbances in relation to age in DS is still limited, and further studies on larger samples are needed.

The positive correlation between BMI and DOES remarks the negative effect of a higher BMI and excessive daytime sleepiness and BMI in both pediatric and adult age [54,55]. As a consequence, a clinical recommendation may be outlined: comorbid risk factors associated with excessive somnolence must always be considered. Further considerations about the possible interconnections with psychopathology are included in the next section.

Our study failed to detect an association between sleep disturbances and nonverbal IQ. Similarly, Joyce and Dimitriou [17] did not observe a relationship between the presence of SBD and cognitive performance in children with DS. On the contrary, Breslin and colleagues [21] found significantly lower verbal IQ in children with DS and comorbid obstructive sleep apnea, whereas, in their study, no differences emerged in the nonverbal IQ. While the effects of sleep disturbances on verbal abilities in children with DS have been reported in several studies [9,22,23,24], the impact of sleep disturbances on nonverbal abilities has not been documented yet. A possible explanation could be the recognized role of sleep-dependent processes of consolidation and integration of declarative information into existing networks [56,57]. Such kind of information is most sensitively detected by verbal IQ measures.

Considering the other neuropsychological variables investigated, a significant correlation between visual-motor integration and DIMS emerged. Suitable visual-motor integration is required for copying movement patterns and images by drawing or handwriting; impaired visual-motor integration is considered a predictor of poor handwriting. Children with DS frequently exhibit poor dexterity and visual-motor integration [58]. The possible negative impact of sleep disturbances on visual-motor integration constitutes a crucial element to take into account for the clinical evaluation and the setting up of therapeutic plans in children with DS.

Sleep disturbances have been demonstrated to significantly affect adaptive behavior in both TD youths [59] and children with neurodevelopmental disorders as autism spectrum disorder [60]. In our sample, we did not observe any association between sleep disturbances and adaptive abilities. Actually, the literature does not provide consistent results about such association in children with DS. For instance, Breslin and colleagues [21] did not find any relation between the presence of obstructive sleep apnea and adaptive behavior standard scores; similarly, Nixon and colleagues [61] reported that the only adaptive domain that significantly correlated with SBD in children with DS was the conceptual score of ABAS-II. In contrast, Brooks and colleagues [62] observed a significant association between the amount of time in slow-wave sleep and adaptive abilities assessed by means of the Vineland Adaptive Behavior Scales. Similarly, another study documented a significant influence of sleep anxiety and parasomnias on the accomplishment of life habits in children with DS [31]. Further research is required to elucidate the impact of sleep problems other than SBD in adaptive abilities of children and adolescents with DS using larger samples.

### 4.2. Association with Psychopathology and Behavioral Problems

Our data revealed a significant correlation between DIMS and externalizing problems. Indeed, the strongest associations emerged with the Attention Problems scale of CBCL and with almost all CPRS scales, in particular with those related to externalizing symptoms. The correlation detected between DIMS and the Irritability/Agitation/Crying and the Hyperactivity/Noncompliance scales of ABC further confirmed such results. SWTD and SDSC Total score exhibited a similar trend, with the strongest association detected with the Attention subscale of CBCL and the Global index—Restless-impulsive, Emotional Lability, and CPRS Global of CPRS; in addition, we found SDSC Total score significantly related with Oppositional, DSM-IV Hyperactive/impulsive and DSM-IV Total scores of CPRS. The significant association between DIMS and parenting difficulties related to the child’s behavior, specifically the relation with the DC scale of PSI-SF, further corroborated the relationship between DIMS and child’s behavioral problems.

These findings are in line with the literature showing that ADHD is frequently coincident with sleep disorders. In individuals with ADHD, the prevalence of sleep disturbances, objectively or subjectively assessed, is reported to range between 25–55% [63,64,65,66]. Difficulties in initiating and maintaining sleep, together with SWTD, have been indicated as sleep disorders frequently occurring in the ADHD population [67,68]. Moreover, DIMS have been previously related with externalizing symptoms in autism spectrum disorder [69], as well in the TD population, where externalizing problems seem to mediate the relationship between DIMS and poor academic performance [70]. A number of studies investigated the role of sleep problems as potential predictive factors of ADHD symptomatology, mainly focusing on the effect of sleep deprivation, SDB, and circadian rhythm disturbances on ADHD symptoms [71]. Some studies suggested that sleep-deprived children might have an intrinsic susceptibility to ADHD; similarly, it has been proposed that ADHD could share a common genetic susceptibility with SBD and circadian rhythm disturbances [71]. However, the association between ADHD symptomatology and sleep disturbances remains complex, and further research is required to disentangle the nature of such a relationship.

Altogether, our results confirm and extend previous findings on the association between externalizing symptoms and sleep disturbances in youths with DS. For instance, parent-reported sleep disturbances in DS have been associated with more attention-deficit/hyperactivity problems [13]; similarly, parent-reported poor sleep quality, evaluated through the Parasomnia subscale of the Children’s Sleep Habits Questionnaire [72], has been found to be predictive of increased daytime hyperactivity/impulsivity [33]. In children with DS, symptoms of attention-deficit/hyperactivity disorder occur at rates 2–3 times higher compared with children with intellectual disability [73,74,75]. Therefore, the identification of the co-occurring factors is crucial for adequate treatment. Given the correlational nature of the present study, it is not clear whether sleep disturbances worsen hyperactivity and inattention symptoms or, in fact, sleep disturbances occur as a consequence of the underlying psychopathological disorder. By studying the longitudinal course of sleep problems, it could be possible to discriminate if hyperactivity/inattention symptoms are a consequence of sleep disturbances in DS or if they precede and worsen this kind of symptomatology.

Parent-reported SBD was found not related per se with psychopathological symptoms in our sample with DS. This is in line with previous studies, which failed to detect significant differences in parent ratings of ADHD symptoms between children with and without obstructive sleep apnea [21]. Intriguingly, Joyce and Dimitrou [17] reported multiple associations between SBD and behavioral problems in TD preschoolers but not in preschoolers with DS. The authors hypothesized that the complex DS phenotype may have introduced some confounding factor masking the effects of sleep in their sample. Beyond phenotypic complexity, another factor to take into account is the peculiarity of DS neurobiology and its possible role in mediating the effects of sleep disturbances in both cognition and behavior. For instance, Spanò and colleagues [76] documented a differential effect of naps on cognition among TD children and children with DS: the authors showed that naps support knowledge acquisition in young TD children, but not in children with DS, where naps result in knowledge loss. Our data further support this unexpected lack of relation between SBD and behavioral and neuropsychological features in children with DS, despite the significantly high prevalence of this kind of sleep disturbance in the DS population. Future research needs to be conducted to better clarify the neurobiological mechanisms mediating the impact of SBD in DS.

We also documented a significant association between internalizing symptoms and sleep problems in our sample, namely Anxiety/Depression and Total Problems subscales of CBCL and DOES. In addition to the abovementioned relationship with BMI, our results seem in line with literature indicating a stronger association of excessive daytime sleepiness in pediatric age with obesity and parent-reported anxiety/depression than the expected association between excessive daytime sleepiness and SDB or objective sleep disruption per se [54]. The relation between internalizing symptoms and DOES could be mediated by a circular interaction involving the quality and quantity of sleep and the activation of physiological systems in response to stress, which may result in further fatigue [70,77]. However, it must be underlined that, in our group, only 4.2% and 12.7% of the youths obtained clinical and borderline scores, respectively, for the DOES scale. Therefore, this limited representation of the clinical population for DOES in our sample should make us consider our results with caution.

Limitations to this work include the absence of measures to assess the verbal abilities of participants. This was because the majority of our patients exhibited significant language impairment that prevented conducting a complete verbal evaluation for all participants. Future studies should examine the associated features of sleep disturbances in cohorts of children and adolescents, including broader phenotypes of DS, as verbal children with DS, and deeper characterization of other aspects, as medical comorbidities. Moreover, we exploited a mono-method approach relying only on parent reports of sleep disturbances: although the instrument we used is well-validated and frequently used in research, objective assessment of sleep would have strengthened our findings. This is particularly true for SBD: parent-report measures of sleep demonstrate poor validity with polysomnography [21], which is the gold standard for the assessment of sleep problems. Therefore, comparative studies using objective measures such as actigraphy or polysomnography for further research are highly desirable. We also did not include a control group in our study. Future work will need to determine the prevalence and correlates of sleep disturbances in children and adolescents with DS in comparison with the TD population and other neurodevelopmental disorders.

Despite these limitations, our results provide important new information on different sleep problems and their behavioral correlates in children with DS, with relevant implications for clinical practice. The use of SDSC allowed us to investigate the prevalence of a range of sleep disorders beyond the most studied SBD. To the best of our knowledge, this is the first study offering an in-depth characterization of sleep problems that most frequently occur in developmental age in an Italian sample of youths with DS. The large range of neuropsychological and psychopathological measures allowed us to dissect the individual and clinical correlates associated with sleep disorders, corroborating the documented relation between DIMS and externalizing symptoms, as well as the association between DOES and internalizing symptoms in children and adolescents with DS. Moreover, we have pointed out, for the first time, the negative association between DIMS and visual-motor integration, as well as the relationship between DOES and BMI in youths with DS. The proper identification of specific sleep disturbances in the DS population could support the process of clinical evaluation, in particular for psychopathological aspects. It is well recognized; indeed, those sleep disturbances are important elements that can mediate psychopathology and cognition. Here, we provided a detailed characterization of these problems in relation to several features of youths with DS. Therefore, it is supposable that handling sleep problems in youths with DS may lead to amelioration of emotional and behavioral outcomes.

## 5. Conclusions

Our results mirror the complex liaisons between sleep, cognition, and mental health. This study focused on individual and behavioral features associated with different kinds of sleep disturbances, including, but not limited to, SBD. First, we focused on the prevalence of sleep disorders in youths with SD, and our results indicated that SBD and DIMS were the most frequently occurring sleep problems in our group of youths, followed by DA and SWTD, whereas DOES and SHY were the less represented sleep disorders in our sample. The second aim of the work was the detailed description of cognitive and behavioral features associated with specific profiles of sleep problems in such a population. Our findings indicate that there is an association between sleep disturbances, mainly disorders in initiating and maintaining sleep, and neurocognitive and behavioral features among children with DS, such as visual-motor integration and externalizing behavior. Moreover, the association of age with DIMS and DOES in our sample paves the road to further investigations aiming at characterizing the developmental trajectories of sleep problems in DS. Sleep disturbances are often not the only comorbidity of psychopathological disorders but also a prodromal symptom. Further work is necessary to explore the bidirectional relationship between psychopathology and sleep disturbances in a larger sample of children and adolescents with DS as well as the neurobiological mechanisms mediating the impact of sleep problems in DS.

## Figures and Tables

**Table 1:** Association between emotional problems and sleep disturbances. Anx. Anxious/depressed; Som. Compl. Somatic Complaints; Aggress. Aggressive Behavior; Intern. Internalizing Behavior; Extern. Externalizing Behavior. * *p* < 0.05; ** Survived after Bonferroni correction *p* ≤ 0.001.

	DIMS *r* (*p*)	SBD *r* (*p*)	DA *r* (*p*)	SWTD *r* (*p*)	DOES *r* (*p*)	SHY *r* (*p*)	TOTAL *r* (*p*)
Anx./Dep.	0.182 (0.137)	0.060 (0.629)	0.109 (0.375)	0.052 (0.675)	0.420 ** (0.000)	−0.059 (0.634)	0.199 (0.105)
Som. Compl.	0.246 * (0.043)	0.201 (0.086)	0.330 * (0.006)	0.336 * (0.005)	0.364 * (0.002)	−0.001 (0.991)	0.376 * (0.002)
Attention	0.486 ** (0.000)	0.183 (0.135)	0.124 (0.080)	0.384 ** (0.001)	0.207 (0.090)	0.166 (0.177)	0.467 ** (0.000)
Aggress.	0.186 (0.129)	0.037 (0.767)	−0.054 (0.661)	0.163 (0.183)	0.074 (0.546)	0.137 (0.267)	0.191 (0.118)
Intern.	0.230 (0.060)	0.160 (0.193)	0.159 (0.195)	0.107 (0.384)	0.351 * (0.003)	0.011 (0.929)	0.291 * (0.016)
Extern.	0.346 * (0.004)	0.159 (0.195)	0.119 (0.335)	0.309 * (0.010)	0.334 * (0.005)	0.084 (0.493)	0.368 * (0.002)
Total Problems	0.373 * (0.002)	0.163 (0.184)	0.180 (0.143)	0.247 * (0.043)	0.403 ** (0.001)	0.040 (0.749)	0.380 ** (0.001)

**Table 2:** Association between sleep problems and symptoms of attention-deficit/hyperactivity disorder. * *p* < 0.05; ** Survived after Bonferroni correction *p* ≤ 0.001.

	DIMS	SBD	DA	SWTD	DOES	SHY	TOTAL
	*r* (*p*)	*r* (*p*)	*r* (*p*)	*r* (*p*)	*r* (*p*)	*r* (*p*)	*r* (*p*)
Oppositional	0.539 **	0.085	0.261	0.376 *	0.424 *	0.265	0.527 **
	0	−0.548	(0–061)	−0.006	−0.002	−0.057	0
Cognitive problems/inattention	0.519 **	0.096	0.279 *	0.320 *	0.309 *	0.122	0.460 *
	0	−0.5	−0.045	−0.021	−0.026	−0.388	−0.001
Hyperactive	0.476 **	0.02	0.264	0.400 *	0.281 *	0.266	0.427 *
	0	−0.886	−0.058	−0.003	−0.044	−0.057	−0.002
Anxious	0.499 **	−0.068	0.251	0.141	0.448 *	−0.072	0.389 *
	0	−0.633	−0.072	−0.32	−0.001	−0.61	−0.004
Perfectionism	0.409 *	0.053	0.319 *	0.161	0.329 *	0.17	0.380 *
	−0.003	−0.708	−0.021	−0.255	−0.017	−0.228	−0.005
Social problems	0.492 **	−0.104	0.149	0.177	0.263	0.1	0.371 *
	0	−0.464	−0.291	−0.408	−0.06	−0.48	−0.007
Psychosomatic	0.231	0.167	0.323 *	0.117	0.27	−0.068	0.291 *
	0.1	−0.236	−0.019	−0.408	−0.053	−0.633	−0.036
ADHD index	0.497 **	0.096	0.278 *	0.370 *	0.306 *	0.157	0.453 *
	0	−0.498	−0.046	−0.007	−0.027	−0.267	−0.001
Global index- Restless-impulsive	0.572 **	0.049	0.301 *	0.474 **	0.329 *	0.181	0.497 **
	0	−0.73	−0.03	0	−0.017	−0.199	0
Emotional lability	0.532 **	0.214	0.256	0.421 **	0.391 *	0.348 *	0.557 **
	0	−0.128	−0.066	0	−0.004	−0.012	0
CPRS	0.588 **	0.104	0.323 *	0.477 **	0.356 *	0.249	0.542 **
Global index	0	−0.462	−0.019	0	−0.01	−0.075	0
DSM-IV Inattentive	0.489 **	−0.041	0.227	0.305 *	0.295 *	0.195	0.421 *
	0	−0.772	−0.106	−0.028	−0.034	−0.166	−0.002
DSM-IV Hyperactive/impulsive	0.526 **	0.066	0.343 *	0.402 *	0.451 *	0.196	0.492 **
	0	−0.641	−0.013	−0.003	−0.001	−0.163	0
DSM-IV Total	0.551 **	0.014	0.299 *	0.375 *	0.375 *	0.189	0.482 **
	0	−0.919	−0.031	−0.006	−0.006	−0.178	0

**Table 3:** Association between sleep disturbances and behavioral problems. * *p* < 0.05; ** Survived to Bonferroni correction *p* ≤ 0.001.

	DIMS	SBD	DA	SWTD	DOES	SHY	TOTAL
	*r* (*p*)	*r* (*p*)	*r* (*p*)	*r* (*p*)	*r* (*p*)	*r* (*p*)	*r* (*p*)
Irritability/Agitation/Crying	0.468 **	0	0.163	0.184	0.262	0.173	0.342 *
	0	−0.998	−0.238	−0.182	−0.055	−0.21	−0.011
Lethargy/	0.234	−0.070	0.304 *	0.018	0.121	−0.078	0.127
Social Withdrawal	−0.088	−0.617	−0.026	−0.9	−0.383	−0.574	−0.36
Stereotypic	0.393 *	−0.057	0.362 *	0.167	0.241	0.05	0.332 *
Behavior	−0.003	−0.681	−0.007	−0.227	−0.079	−0.72	−0.014
Hyperactivity/	0.428 **	0.021	0.076	0.193	0.2	0.074	0.269 *
Noncompliance	−0.001	−0.88	−0.585	−0.162	−0.147	−0.594	−0.049
Inappropriate Speech	0.288 *	0.026	0.053	0.027	0.048	−0.129	0.129
	−0.035	−0.851	−0.701	−0.846	−0.732	−0.352	−0.353
ABC Total Score	0.478 **	−0.027	0.246	0.165	0.244	0.042	0.317 *
	0	−0.847	−0.073	−0.233	−0.075	−0.761	−0.02

## Data Availability

The data presented in this study are available on request from the corresponding author.
